# Total synthesis of 1-oxomiltirone via Suzuki coupling

**DOI:** 10.1007/s13659-013-0034-7

**Published:** 2013-06-05

**Authors:** Chun-Miao Li, Hui-Chun Geng, Ming-Ming Li, Gang Xu, Tie-Jun Ling, Hong-Bo Qin

**Affiliations:** 1 State Key Laboratory of Phytochemistry and Plant Resources in West China, Kunming Institute of Botany, Chinese Academy of Sciences, Kunming, 650201 China; 2 University of Chinese Academy of Sciences, Beijing, 100049 China; 3 Key Laboratory of Tea Biochemistry and Biotechnology of Ministry of Education & Ministry of Agriculture, Anhui Agricultural University, Hefei, 230036 China

**Keywords:** total synthesis, Suzuki coupling, 1-oxomiltirone, abietane diterpenes, versatile intermediate

## Abstract

Abietane diterpenes and miltirone series have shown important activities and for medical purposes in order to achieve the total synthesis of 1-oxomiltrone **1** and miltirone **4**, a versatile intermediate **6** was found. The compound **6** could be used as a precursor A-B-C rings with different oxidative degrees in selected abietane diterpenes when synthesized through high yield Suzuki reaction and subsequent cyclization, and total synthesis of 1-oxomiltirone (**1**) has been achieved.

**Electronic Supplementary Material:**

Supplementary material is available for this article at 10.1007/s13659-013-0034-7 and is accessible for authorized users.

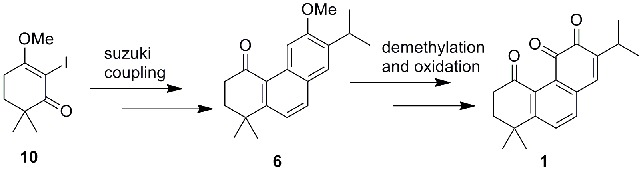

## Introduction

The miltirone series, **1**, **3** and **4** have shown to possess anti-tumor effects,[1] with pisiferic acid **5** (Figure 1) shown to exhibit anti-microbial activity, specifically against all gram positive bacteria.[2] Due to their important activities, research has focused on the total syntheses of these compounds.[3–5] In our endeavor to modify bioactive abietane diterpenes for medical purposes, we found that compound **6** was a key intermediate to accomplish total synthesis of xanthoperyl methyl ether **2** and pisiferic acid **5**.[3] It has several characteristics: 1) A ring can be reduced to cyclohexane, as with pisiferic acid **5**. 2) B ring can be oxidized into ortho-quinone, as with xanthoperyl methyl ether **2**. Furthermore, methoxy group in C ring can be transformed into *ortho*-quinone by demethylation of the methyl ether and subsequent oxidation.[4] We concluded that **6** could be a precursor of a A-B-C ring with different oxidative degree in the abietane diterpenes. Accordingly, the miltirone series of compounds could be synthesized through a key intermediate **6**.

In the Mukeherjee's report,[6] ketone **6** was synthesized in nine steps from 2-isopropyl phenyl methyl ether through to a C-B-A sequence. Alternatively, the same skeleton of **6** could be constructed through annulation strategy of 2-aryl dienone reported by Majetich[5]. This was derived from an aromatic metal nucleophile ring-opening of silyl enol epoxide, and a vinyl addition with a subsequent PDC oxidative re-arrangement. This strategy produced low yields in steps such

**Figure 1.** Selected diterpenes which could be synthesized from intermediate **6**

as twice ketone $\alpha$-methyl introduction and oxidative rearrangement. This report therefore, will describe an efficient synthesis of 2-aryl dienone via Suzuki reaction and demonstrate its synthetic utility by total synthesis of 1-oxomiltirone.

## Results and Discussion

Our retrosynthesis as outlined in Scheme **1** displays the possible tricycle skeleton that could be formed by sequential vinyl addition of **9**, Friedel-Crafts cyclization and base induced aromatization.[6] The key intermediate **9** could be

*To whom correspondence should be addressed. E-mail: qinhongbo@mail.kib.ac.cn (H.B. Qin); lingtj@ahau.edu.cn (T.J. Ling)

synthesized from the Suzuki reaction of vinyl iodide **10** and aromatic boronic acid **11**, which could be transformed from known enol methyl ether ketone **12** and aromatic bromide **13** (see Electronic Supplementary Material).

**Scheme 1.** Retrosynthetic analysis of **6**

Synthesis of the coupling component iodide **10** was carried out with $I_2$ and tri-methyl orthoformate,[7] whilst under standard condition ($I_2$, Py.) failed to give iodide **10**. It was then coupled with aromatic boronic acid to derive ketone **9** catalyzed by $PdCl_2(PhCN)_2$,[8] which could be used without purification. After treatment with vinyl magnesium bromide in an ice-water bath and acid workup, 2-aryl-dienone **8** was obtained in a 53% isolation yield. Cyclization with $BF_3{\cdot}OEt_2$ and *t*-BuOK induced an aromatization which gave rise to key intermediate **6** (Scheme 2).

**Scheme 2.** Synthesis of tricyclic intermediate **6**

To demonstrate its synthetic utility, the total synthesis of 1-oxomiltirone was achieved from **6** via $BBr_3$ de-methylation of the methyl ether and oxidation by Dess-Martin periodinane with a yield of 64%, of which $^1$H and $^{13}$C NMR spectra were totally equal with the reported data (Scheme 3).[9]

Furthermore, the additional application could synthesize miltirone by the reduction of the ketone group in **6**,[5] de-methylation of the methyl ether and the subsequent Dess-Martin oxidation.[4]

In conclusion, we have developed a short synthesis route to versatile intermediate **6** via Suzuki coupling as a key reaction. Its synthetic utility was demonstrated by the synthesis of 1-oxomiltirone.

**Scheme 3.** Synthesis of 1-oxomiltirone **1**

## Experimental Section

**General Experimental Procedures.** All reactions were performed with dry solvents under anhydrous conditions, unless otherwise noted. Dry solvent was obtained by a standard procedure. Dry tetrahydrofuran (THF) was distilled over a sodium-potassium alloy. Dichloromethane was distilled over calcium hydride. Yields refer to chromatographically and spectroscopically homogeneous materials, unless otherwise noted. Reagents were used as received without further purification, unless otherwise stated. Silica gel (200–300 mesh), was supplied by Qingdao Marine Chemical Co., Ltd., China), and light petroleum ether (bp 60–90 ºC), and ethyl acetate (EA) were used for product purification by flash column chromatography. Melting Point (MP) was determined with an X-4 Taike micro melting point apparatus and was uncorrected. NMR spectra were recorded in a $CDCl_3$ solution on a Bruker AV-400 instrument with tetramethylsilane (TMS) as an internal reference. IR spectra were recorded neat or with KBr pellets on a Bruker Tensor 27 FT-IR spectrometer. High-resolution mass spectral analysis (HRMS) data were recorded via electron impact mass spectrometry using a time of flight analyzer.

**Scheme 4.** Synthesis of armomatic boronic acid **11**

**2-(4-Bromo-2-methoxyphenyl)propan-2-ol (17).** A suspension containing compound **15** (5 g, 23.2 mmol) and also potassium carbonate (9.6 g, 69.6 mmol) in anhydrous acetone (46 mL) was added to dimethyl sulfate (2.68 mL, 28.3 mmol), with the reaction mixture stirred at 80 °C for three hours. After the reaction was complete by TLC modification, the mixture was filtrated and the filtrate further concentrated. Water (300 mL) was then added and then the mixture was extracted with ethyl acetate and the organic phase were washed with saturated NaCl, and dried over anhydrous sodium sulfate, filtrated, and concentrated to derive compound **16** as a colorless solid (5.7 g, 24.9 mmol), which was used without further purification.

Methyl magnesium iodide was prepared as follows: Methyl iodide (2.00 mL, 32 mmol) in ether (10 mL) was added in a dropwise manner to a suspension of magnesium turnings (770

mg, 32 mmol) in ether (10 mL). When the magnesium turnings disappeared after stirring for a while, a solution of **16** (5.7 g, 24.9 mmol) in dry THF (15 mL) was added slowly and the reaction completed after 1 h stirring at r.t. monitored by TLC. The reaction was quenched with saturated ammonium chloride, the mixture was extracted with EA (3 × 15 mL). The organic layer was washed with brine (1 × 15 mL) and dried over anhydrous $Na_2SO_4$. The crude product was purified by silica gel column chromatography to give **17** (4.64 g, 81.6%) as a pale yellow oil. $^1$H NMR (400 MHz, $CDCl_3$) $\delta_H$ ppm: 7.20 (d, $J$ = 8.2 Hz, 1H), 7.08 (dd, $J$ = 8.4, 1.6 Hz, 1H), 7.04 (d, $J$ = 1.6 Hz, 1H), 3.91 (s, 3H), 1.57 (s, 6H).

**4-Bromo-1-isopropyl-2-methoxybenzene (13).** To a solution of **17** (2.6 g, 10.6 mmol) dissolved in dichloromethane (50 mL) at 0 °C, triethylsilane (3.4 mL, 21.1 mmol) was added, followed by boron tri-fluoride diethyl etherate (1.06 mL, 10.6 mmol). The solution was allowed to warm to room temperature and stirred for 1 h. Subsequently, it was quenched with saturated aqueous $Na_2CO_3$ solution. The aqueous phase was acidified with 1N HCl and extracted with EA (3 × 15 mL), with the organic phase washed with saturated $NaHCO_3^-$, dried over anhydrous sodium sulfate, filtered, concentrated and purified by column chromatography (PE) to extract compound **13** (2.06 g, 84.8%) as a colorless oil. $^1$H NMR (400 MHz, $CDCl_3$) $\delta_H$ ppm: 7.05 (s, 2H), 6.96 (s, 1H), 3.82 (s, 3H), 3.25 (m, $J$ = 6.8 Hz, 1H), 1.18 (d, $J$ = 6.8 Hz, 6H); $^{13}$C NMR (100 MHz, $CDCl_3$) $\delta_C$ ppm: 157.4, 136.1, 127.3, 123.4, 119.5, 113.7, 55.5, 26.4, 22.4, 22.4.

**Armomatic boronic acid (11).** A solution of **13** (2.84 g, 12.4 mmol) in THF (51 mL) was cooled to – 78 °C and *n*-BuLi (6 mL of 2.5 M in hexane) was added. The mixture was stirred at this temperature for 40 min, to which tri-isopropyl borate (4.32 mL, 18.6 mmol) was added, and then the mixture was gradually warmed to – 30 °C. The reaction was quenched with 3M HCl (20 mL) and diluted with EA (30 mL), with the organic phase collected. The aqueous phase was extracted with EA (3 × 30 mL). All organic layers were combined, washed with brine (30 mL), dried ($Na_2SO_4$), filtered, concentrated and purified by column chromatography (PE/EA = 4/1) to extract compound **11** as a white solid (1.76 g, 73.3 %) (mp 141–144 °C). $^1$H NMR (400 MHz, $CDCl_3$) $\delta_H$ ppm: 7.06 (s, 2H), 6.97 (s, 1H), 3.82 (s, 3H), 3.25 (m, 1H), 1.18 (d, $J$ = 6.8 Hz, 6H); $^{13}$C NMR (100 MHz, $CDCl_3$) $\delta_C$ ppm: 156.4, 142.2, 128.4, 125 .8, 116.3, 116.3, 55.3, 26.9, 22.5, 22.5.

**2-Iodo-3-methoxy-6,6-dimethylcyclohex-2-enone (10).** A mixture of keto compound **12** (959 mg, 6.22 mmol) and iodine (3.16 g, 12.4 mmol) in tri-methylortho-formate (32 mL) was stirred at room temperature for 12 h. After completion of the reaction as indicated by TLC, the reaction mixture was diluted with EA (10 mL) and the organic layer was washed with saturated aqueous $Na_2S_2O_3$ (2 × 30 mL) and brine (1 × 60 mL). The organic layer was dried over anhydrous $Na_2SO_4$ and concentrated *in vacuo*. The residue was purified using silica gel column chromatography (PE/EA = 50/1) to derive compound **10** as a white solid (1.03 g, 58.9%) (mp 91–93 °C). $^1$H NMR (400 MHz, $CDCl_3$) $\delta_H$ ppm: 3.93 (s, 3H), 2.70 (t, $J$ = 6.4 Hz, 2H), 1.86 (t, $J$ = 6.4 Hz, 2H), 1.14 (s, 6H); $^{13}$C NMR (100 MHz, $CDCl_3$) $\delta_C$ ppm: 204.3, 176.6, 100.8, 55.7, 35.1, 28.2, 26.2, 24.6, 24.6; IR (KBr) $\nu_{max}$ cm$^{-1}$: 2964, 2870, 1734, 1790, 1617, 1578, 1459, 1422, 1366, 1299, 1170, 1103, 1049, 1013, 998, 898.; HRMSEI *m/z*: calcd for $C_9H_{13}IO_2$ $[M]^+$: 279.9960, found 279.9969.

**2-Aryl-dienone (8).** A 100 mL, round-bottomed flask, equipped with a Teflon-coated magnetic stirring bar and an argon inlet adaptor, was charged with 1.0 g (3.57 mmol) of **10**, 1.04 g (5.36 mmol) of (4-isopropyl-3-methoxyphenyl)boronic acid, 1.32 g (5.71 mmol, 1.6 eq) of silver (I) oxide ($Ag_2O$), 0.11 g (0.36 mmol, 10 mol%) of tri-phenylarsine, 0.53 g (0.18 mmol, 5 mol%) of palladium bis(benzonitrile) dichloride, 20 mL of THF and 2.5 mL of water. The reaction mixture, flushed with argon, was stirred for 3 h and then quenched with the addition of 12.5 mL of saturated aqueous ammonium chloride. The aqueous phase was extracted with EA (3 × 20 mL) .The combined organic phases were washed using 60 mL of brine. The resulting organic phase was dried over anhydrous $Na_2SO_4$, filtered, and concentrated at reduced pressure to give the brown oil **9** which was used without further purification in the next step. An analytical sample was obtained by flash chromatography, petroleum ether/ethyl acetate = 100/1–10/1. $^1$H NMR (400 MHz, $CDCl_3$) $\delta_H$ ppm: 7.14 (d, $J$ = 8.0 Hz, 1H), 6.68 (d, $J$ = 7.6 Hz, 1H), 6.63 (s, 1H), 3.79 (s, 3H), 3.72 (s, 3H), 3.28 (m, $J$ = 6.8 Hz, 1H), 2.71 (t, $J$ = 6.4 Hz, 2H), 1.93 (t, $J$ = 6.4 Hz, 2H), 1.20 (d, $J$ = 6.8 Hz, 6H), 1.18 (s, 6H); $^{13}$C NMR (100 MHz, $CDCl_3$) $\delta_C$ ppm: 202.0, 169.9, 156.0, 135.1, 131.9, 125.1, 122.9, 118.5, 113.0, 55.6, 55.3, 39.7, 34.0, 26.5, 24.6, 22.8, 22.7; IR (neat) $\nu_{max}$ cm$^{-1}$: 2961, 2926, 1639, 1614, 1569, 1464, 1447, 1480, 1371, 1349, 1326, 1254, 1221, 1165, 1052, 1026, 963, 894, 881, 854, 816, 790, 778, 767, 747; HRMSEI *m/z*: calcd for $C_{19}H_{26}O_3$ $[M]^+$: 302.1882, found 302.1885.

The crude compound **9** (1.03 g) in THF (30 mL) was treated dropwise at 0 °C with 14.3 mL (14.3 mmol) of vinyl-magnesium bromide (1.0 M solution in THF) over a 10-min period. The reaction mixture was stirred at ambient temperature for 2 h. The reaction mixture was quenched with saturated aqueous ammonium chloride and then 1M HCl (5.2 mL). The aqueous phase was extracted with EA (3 × 30 ml). The combined organic phases were washed successively with 1M HCl and saturated bicarbonate sodium, and then washed with 60 mL of brine. The resultant organic phase was dried over anhydrous $Na_2SO_4$, then filtered, and concentrated at reduced pressure to provide a pale yellow oil. The crude oil was purified using silica gel column chromatography (PE/EA = 50/1) to derive compound **8** as a pale yellow oil (564 mg, 53% in two steps). $^1$H NMR (400 MHz, $CDCl_3$) $\delta_H$ ppm: 7.11 (d, $J$ = 7.6 Hz, 1H), 6.55 (br. d, $J$ = 7.6 Hz, 1H), 6.46 (br. s, 1H), 6.18 (dd, $J$ = 17.6, 12.0 Hz, 1H), 5.18 (dd, $J$ = 12.0, 1.2 Hz, 1H), 5.06 (dd, $J$ = 17.6, 1.2 Hz, 1H), 3.81 (s, 3H), 3.26 (m, $J$ = 6.8 Hz, 1H), 2.62 (t, $J$ = 6.8 Hz, 2H), 1.98 (t, $J$ = 13.6, 7.2 Hz, 2H), 1.32 (s, 6H), 1.19 (d, $J$ = 6.8 Hz, 6H); $^{13}$C NMR (100 MHz, $CDCl_3$) $\delta_C$ ppm: 198.4, 161.8, 156.2, 136.9, 135.4, 134.6, 133.9, 125.3, 122.5, 121.5, 112.5, 55.3, 37.8, 35.4, 34.6, 27.5, 27.5, 26.5, 22.6, 22.6; IR (neat) $\nu_{max}$ cm$^{-1}$: 2961, 2932, 2868, 1673, 1610, 1568, 1504, 1464, 1405, 1351, 1333, 1311, 1276, 1246, 1198, 1172, 1094, 1041, 924, 899, 880, 850, 817, 795, 769, 695, 598; HRMSEI *m/z*: calcd for $C_{20}H_{26}O_2$ $[M]^+$: 298.1933, found 298.1912.

**Key intermediate (6).** A solution of 200 mg of **8** (0.67 mmol) and 436 $\mu$L of $BF_3OEt_2$ (4.36 mmol) in 6.5 mL of $CCl_4$ was refluxed for 2 h. The reaction mixture was diluted with 10 mL of EA and neutralized with 15 mL of saturated aqueous $NaHCO_3$. The aqueous phase was extracted with EA (3 × 15 mL). The combined organic phases were washed with 45 mL of brine. The organic phase was dried over anhydrous $Na_2SO_4$, filtered, and concentrated at reduced pressure to provide the brown oil which was used without further purification in the next step. The crude compound **7** (210 mg, 0.67 mmol) and *t*-BuOK (301 mg, 2.68 mmol) in 6 mL of *t*-butanol was refluxed for 3 h. The reaction mixture was quenched with water and the aqueous phase was extracted with EA (3 × 5 mL). The combined organic phases were washed with 15 mL of brine. The resulting organic phase was dried over anhydrous $Na_2SO_4$, filtered, and concentrated at reduced pressure to give the yellow oil. The crude oil was purified by silica gel column chromatography (PE/EA = 50/1) to afford compound **6** as yellow oil (131 mg, 66% in two steps). $^1$H NMR (400 MHz, $CDCl_3$) $\delta_H$ ppm: 8.87 (s, 1H), 7.89 (d, $J$ = 8.8 Hz, 1H), 7.55 (s, 1H), 7.38 (d, $J$ = 8.8 Hz, 1H), 3.99 (s, 3H), 3.39 (m, 1H), 2.83 (t, $J$ = 6.8 Hz, 2H), 2.07 (t, $J$ = 6.8 Hz, 2H), 1.44 (s, 6H), 1.27 (d, $J$ = 6.8 Hz, 6H); $^{13}$C NMR (100 MHz, $CDCl_3$) $\delta_C$ ppm: 201.2, 158.7, 153.5, 138.4, 134.1, 130.9, 127.9, 124.7, 121.5, 121.1, 104.6, 55.4, 37.3, 36.9, 35.1, 30.3, 30.0, 29.8, 27.0, 22.5.

**1-Oxomiltirone (1).** Boron tri-bromide (4.04 mL of 1.0 M solution in $CH_2Cl_2$) was added to a solution of **6** (150 mg, 0.51 mmol) in $CH_2Cl_2$ (5 mL) at – 78 °C. The mixture was allowed to warm to 0 °C over a period of 2 h. The reaction mixture was quenched with water. The aqueous phase was extracted with chloroform (3 × 5 mL) and the combined organic phases were washed with 15 mL of brine. The resulting organic phase was dried over anhydrous sodium sulfate, filtered, and concentrated at reduced pressure to derive a brown oil **14** which was used without further purification in the next step. Analytical sample was obtained by flash chromatography, petroleum ether/ethyl acetate = 50/1. $^1$H NMR (400 MHz, $CDCl_3$) $\delta_H$ ppm: 9.17 (s, 1H), 7.92 (d, $J$ = 8.6 Hz, 1H), 7.58 (s, 1H), 7.35 (d, $J$ = 8.6 Hz,1H), 3.39 (m, 1H), 2.83 (t, $J$ = 6.8 Hz, 2H), 2.07 (t, $J$ = 7.2 Hz, 2H), 1.44 (s, 6H), 1.27 (d, $J$ = 6.8 Hz, 6H); $^{13}$C NMR (100 MHz, $CDCl_3$) $\delta_C$ ppm: 203.2, 157.2, 154.8, 137.5, 135.3, 131.2, 127.9, 125.3, 123.6, 120.2, 109.1, 36.7, 36.5, 35.1, 29.9, 27.2, 22.5.

The crude compound **14** (150 mg) was dissolved in dichloromethane (12 mL) and Dess-Martin periodinane (560 mg, 1.32 mmol) was added. The solution was stirred overnight at room temperature and then diluted with ethyl acetate. The organic phase was separated and washed with 1 N NaOH, water, and brine, dried over $MgSO_4$, and concentrated *in vacuo*. The crude product was purified using silica gel column chromatography (PE/EA = 20/1) to derive a red solid **1** (100 mg, 64 % in two steps) (mp 68–71 °C). $^1$H NMR (400 MHz, $CDCl_3$) $\delta_H$ ppm: 7.53 (d, $J$ = 7.8 Hz, 1H), 7.33 (d, $J$ = 8.4 Hz, 1H), 7.08(s, 2H), 3.02 (dsp, $J_{15,16}$ = $J_{15,17}$ = 7.2 Hz, 1H), 2.90 (t, 2H), 2.05 (t, 2H), 1.32 (s, 6H), 1.16 (d, $J$ = 7.2 Hz, 6H); $^{13}$C NMR (150 MHz, $CDCl_3$) $\delta_C$ ppm: 198.9, 183.7, 183.1, 153.8, 146.4, 138.0, 137.9, 135.5, 132.7, 132.0, 131.0, 36.5, 36.1, 34.9, 29.1, 26.9, 21.5, 21.5.

## Electronic Supplementary Material

Supplementary material is available in the online version of this article at http://dx.doi.org/10.1007/s13659-013-0034-7 and is accessible for authorized users.

## Acknowledgments

We would like to thank the Programs of "One Hundred Talented People" for its financial support.

**Open Access** This article is distributed under the terms of the Creative Commons Attribution License which permits any use, distribution, and reproduction in any medium, provided the original author(s) and source are credited.

## Electronic supplementary material

Supplementary material, approximately 1.09 MB.
