# Supplementary material for: Total synthesis of 1-oxomiltirone via Suzuki coupling
Source: Nat Prod Bioprospect. 2013 Jun 5;3(3):117–20. doi: 10.1007/s13659-013-0034-7 (PMC4131665; doi:10.1007/s13659-013-0034-7)
Supplement: Supplementary file 1 — Supplementary material, approximately 1.09 MB. [file 13659_2013_34_MOESM1_ESM.pdf]

Electronic Supplementary Material

Total synthesis of 1-oxomiltirone via Suzuki coupling

Chun-Miao LI,[a,c] Hui-Chun GENG,[a,b] Ming-Ming LI,[a] Gang XU,[a] Tie-Jun LING,[c,]* and Hong-Bo QIN[a,]*

[a]State Key Laboratory of Phytochemistry and Plant Resources in West China, Kunming Institute of Botany, Chinese Academy of Sciences, Kunming 650201, China

[b]University of Chinese Academy of Sciences, Beijing 100049, China

[c]Key Laboratory of Tea Biochemistry and Biotechnology of Ministry of Education & Ministry of Agriculture, Anhui Agricultural University, Hefei 230036, China

Received 22 April 2013; Accepted 20 May 2013

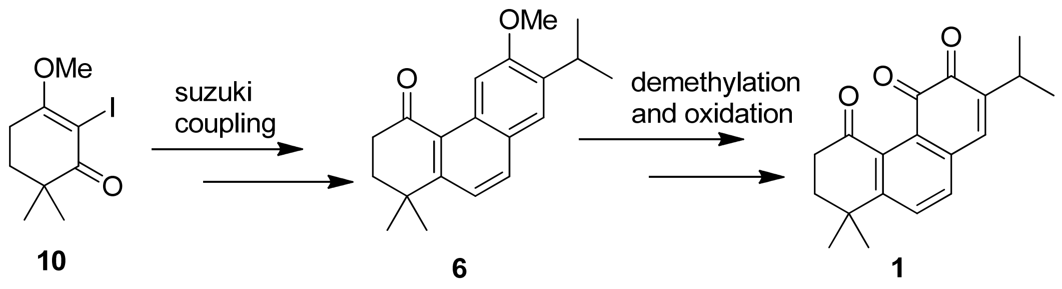

*To whom correspondence should be addressed. E-mail: qinhongbo@mail.kib.ac.cn (H.B. Qin); lingtj@ahau.edu.cn (T.J. Ling)

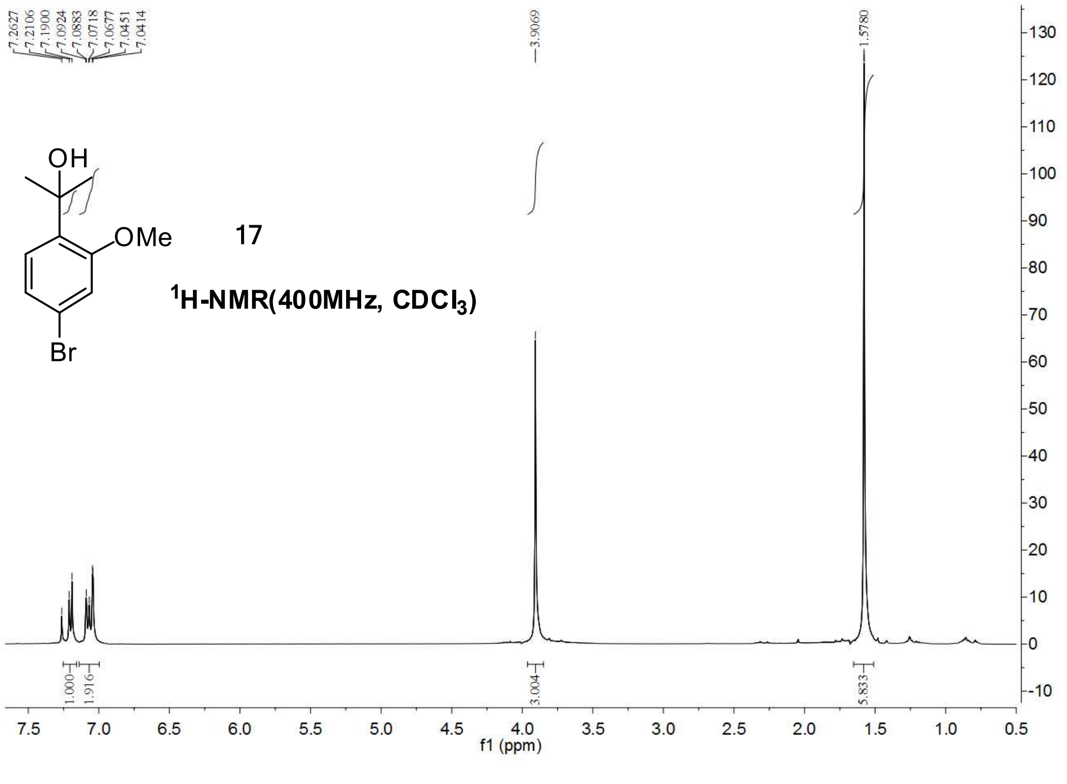

OH
OMe
Br
17
1H-NMR(400MHz, CDCl3)
7.2627
7.2106
7.1900
7.0924
7.0883
7.0718
7.0677
7.0451
7.0414
3.9069
1.5780
1.000
1.916
3.004
5.833
f1 (ppm)

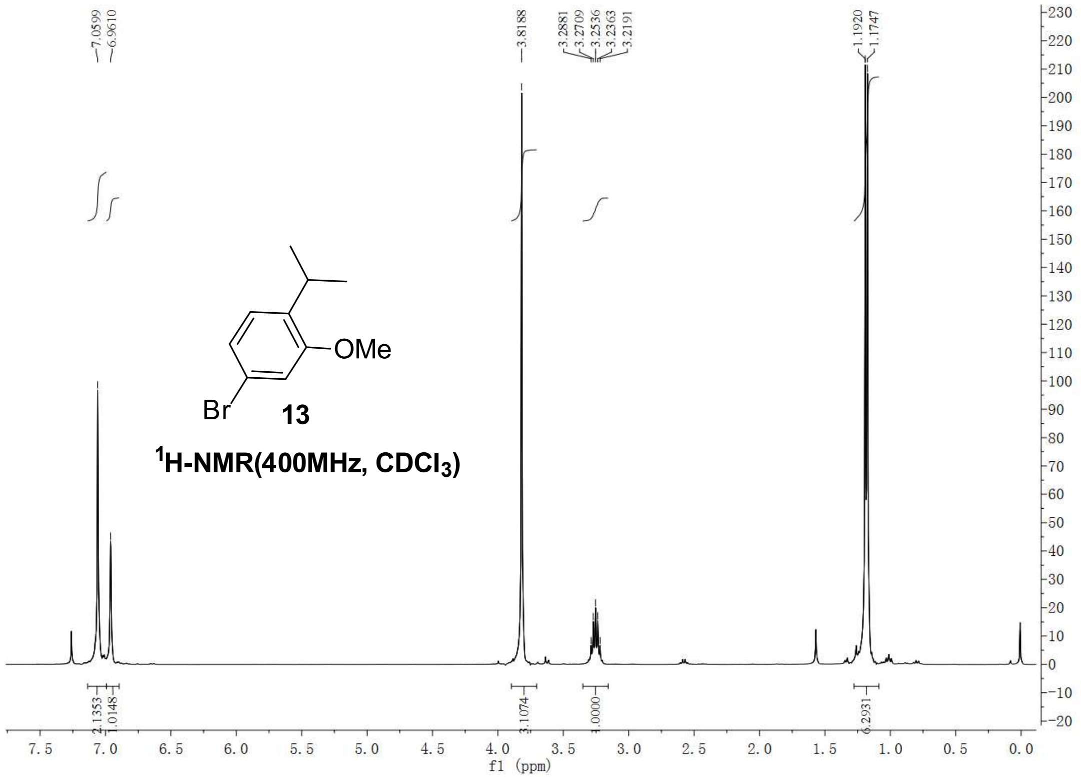

7.0599
6.9610
3.8188
3.2881
3.2709
3.2536
3.2363
3.2191
1.1920
1.1747
OMe
Br
13
1H-NMR(400MHz, CDCl3)
2.1353
1.0148
3.1074
1.0000
6.2931
f1 (ppm)

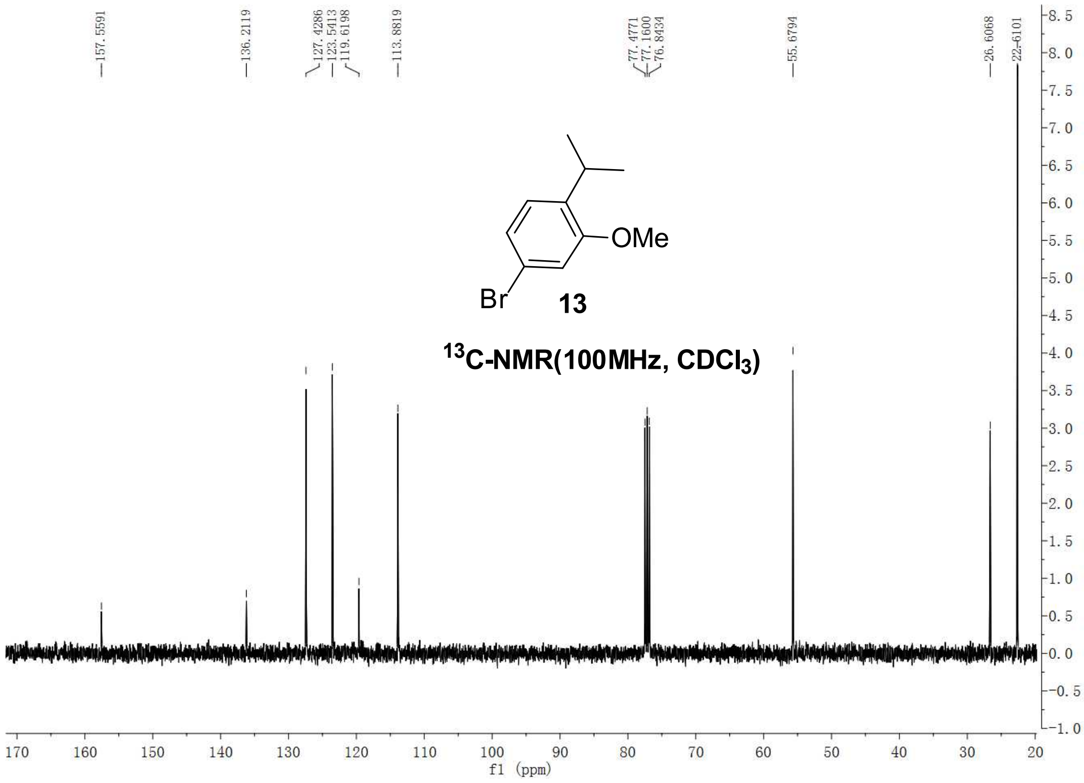

157.5591
136.2119
127.4286
123.5413
119.6198
113.8819
77.4771
77.1600
76.8434
55.6794
26.6068
22.6101
OMe
Br
13
13C-NMR(100MHz, CDCl3)
f1 (ppm)

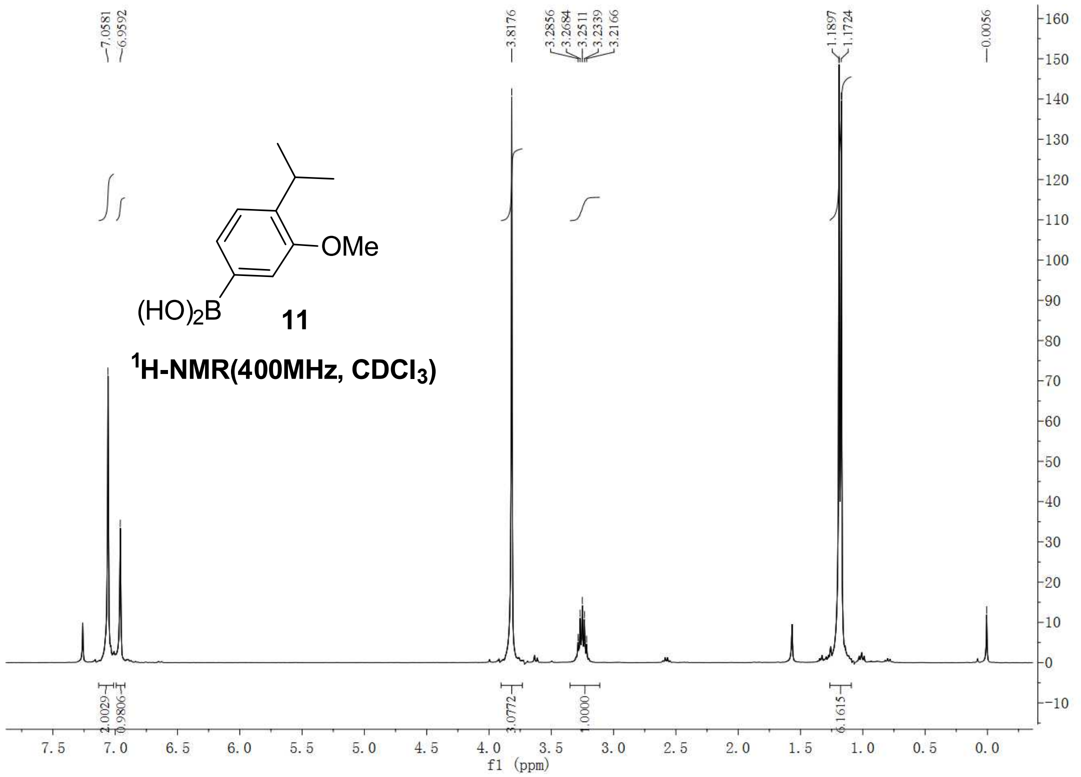
7.0581
6.9592
3.8176
3.2856
3.2684
3.2511
3.2339
3.2166
1.1897
1.1724
0.0056
OMe
(HO)2B
11
1H-NMR(400MHz, CDCl3)
2.0029
0.9806
3.0772
1.0000
6.1615
f1 (ppm)

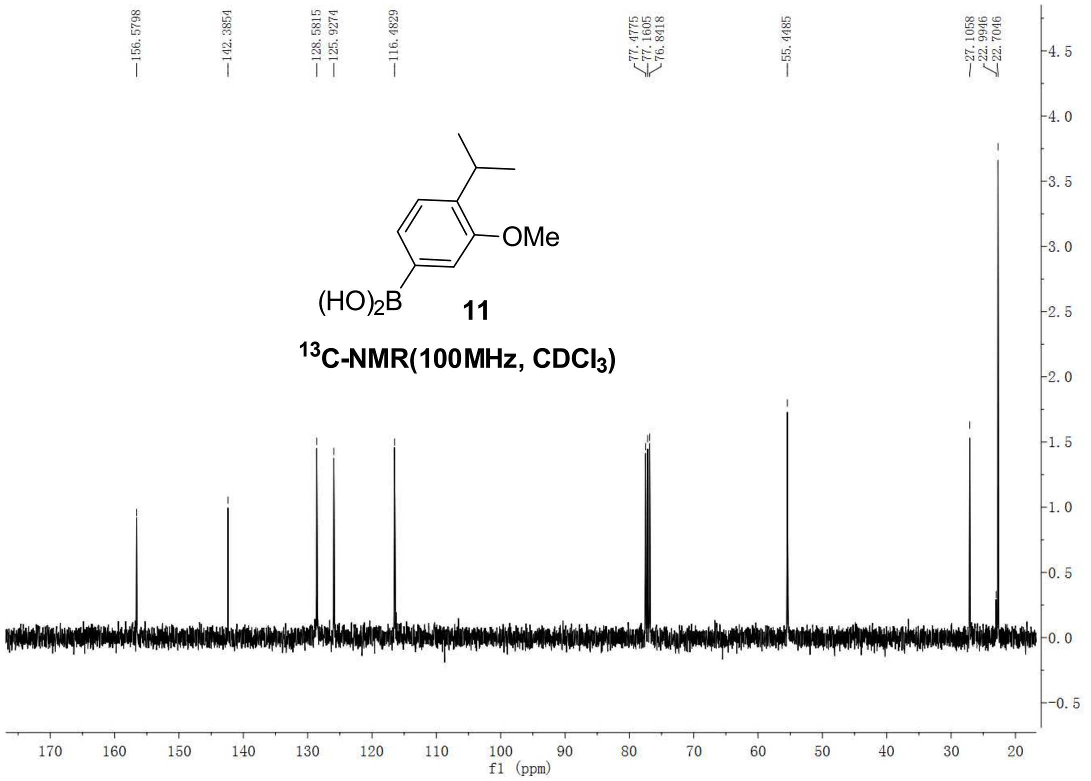
156.5798
142.3854
128.5815
125.9274
116.4829
77.4775
77.1605
76.8418
55.4485
27.1058
22.9946
22.7046
OMe
(HO)2B
11
13C-NMR(100MHz, CDCl3)
f1 (ppm)

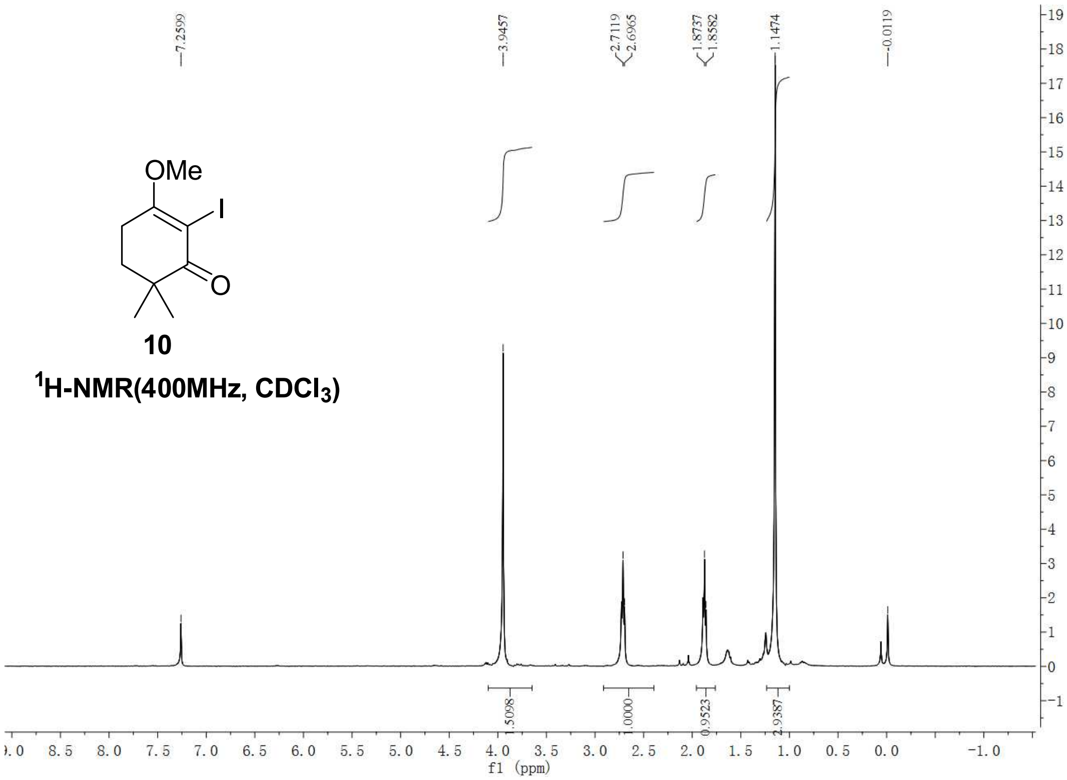
OMe
I
O
10
^{1}H-NMR(400MHz, CDCl$_3$)
7.2599
3.9457
2.7119
2.6965
1.8737
1.8582
1.1474
-0.0119
1.5098
1.0000
0.9523
2.9387
f1 (ppm)

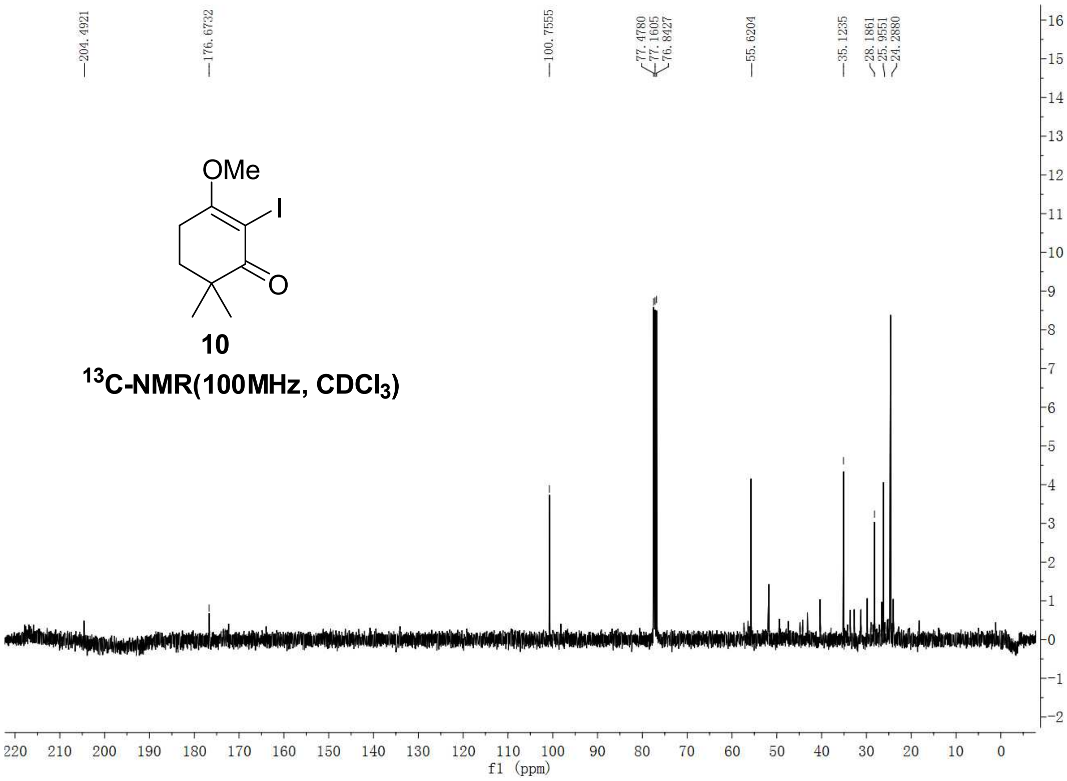
OMe
I
O
10
^{13}C-NMR(100MHz, CDCl$_3$)
204.4921
176.6732
100.7555
77.4780
77.1605
76.8427
55.6204
35.1235
28.1861
25.9551
24.2880
f1 (ppm)

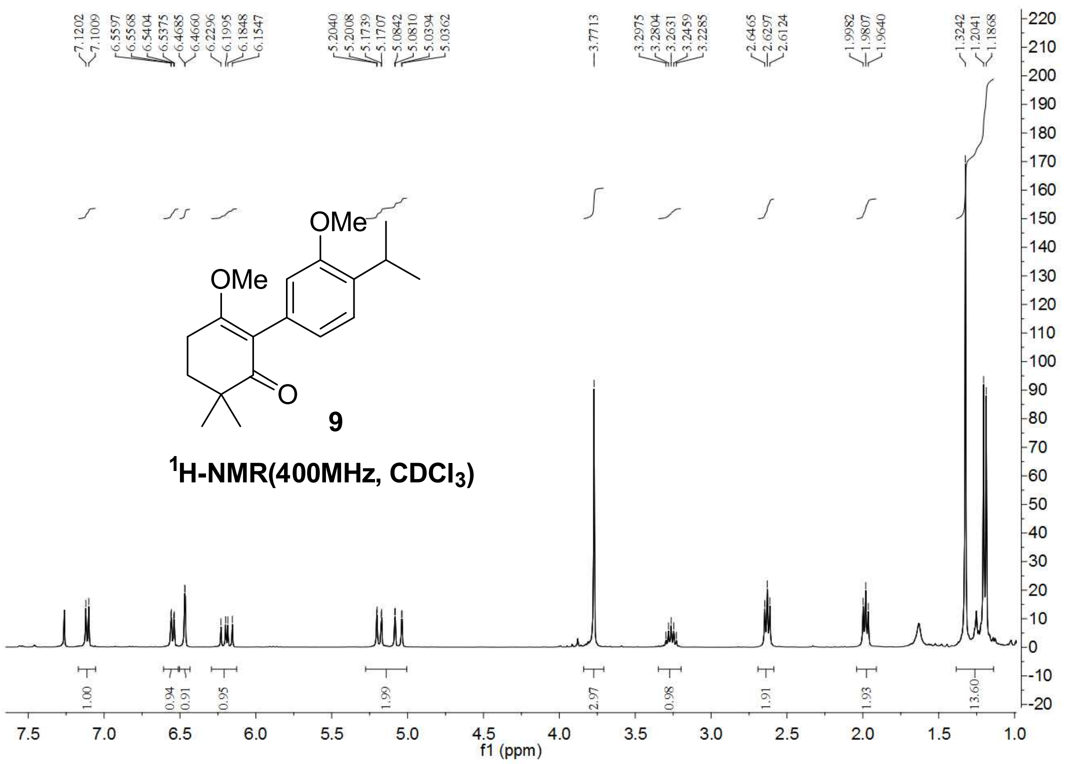
OMe
OMe
O
9
^{1}H-NMR(400MHz, CDCl$_3$)
7.1202 7.1009 6.5597 6.5568 6.5404 6.5375 6.4685 6.4660 6.2296 6.1995 6.1848 6.1547
5.2040 5.2008 5.1739 5.1707 5.0842 5.0810 5.0394 5.0362
3.7713
3.2975 3.2804 3.2631 3.2459 3.2285
2.6465 2.6297 2.6124
1.9982 1.9807 1.9640
1.3242 1.2041 1.1868
1.00 0.94 0.91 0.95 1.99 2.97 0.98 1.91 1.93 13.60
f1 (ppm)

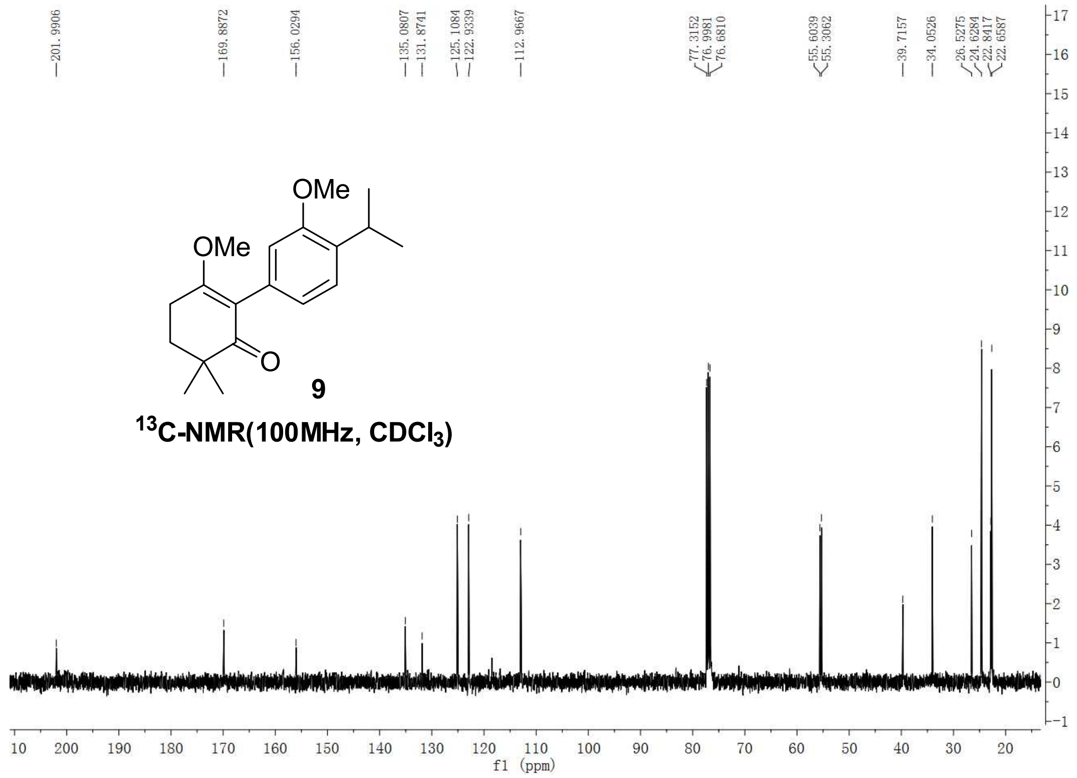
OMe
OMe
O
9
^{13}C-NMR(100MHz, CDCl$_3$)
201.9906
169.8872
156.0294
135.0807 131.8741
125.1084 122.9339
112.9667
77.3152 76.9981 76.6810
55.6039 55.3062
39.7157
34.0526
26.5275 24.6284 22.8417 22.6587
f1 (ppm)

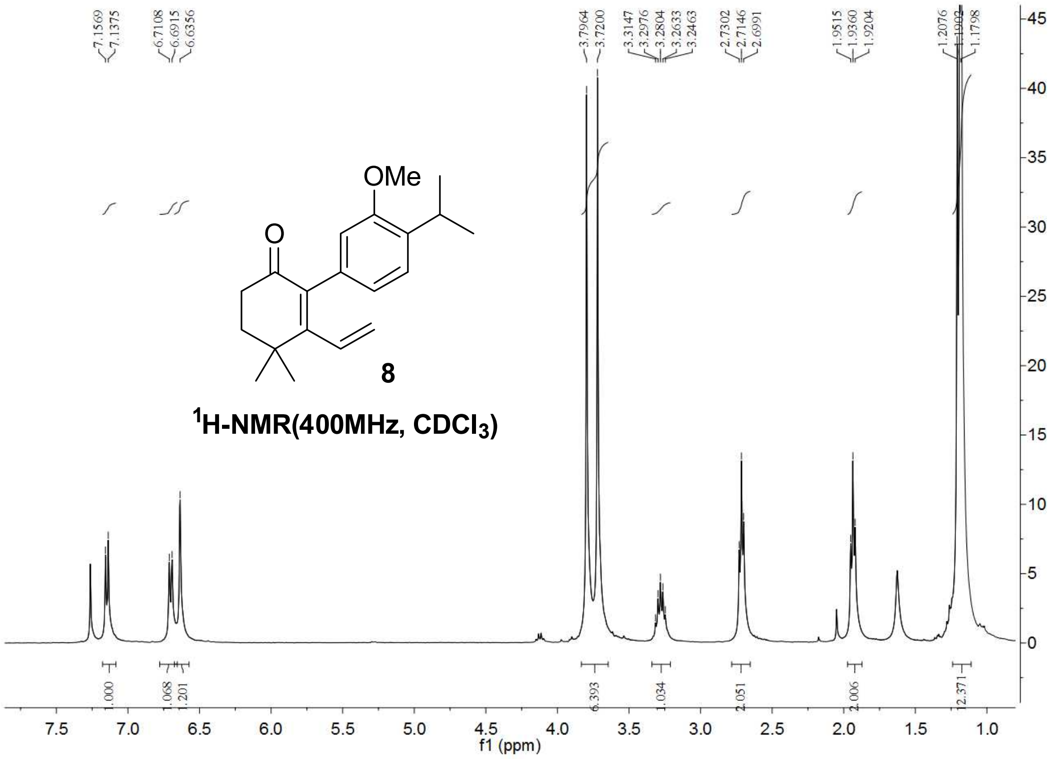

7.1569
7.1375
6.7108
6.6915
6.6356
3.7964
3.7200
3.3147
3.2976
3.2804
3.2633
3.2463
2.7302
2.7146
2.6991
1.9515
1.9360
1.9204
1.2076
1.1798
OMe
O
8
^{1}H-NMR(400MHz, CDCl$_3$)
1.000
1.068
1.201
6.393
1.034
2.051
2.006
12.371
f1 (ppm)

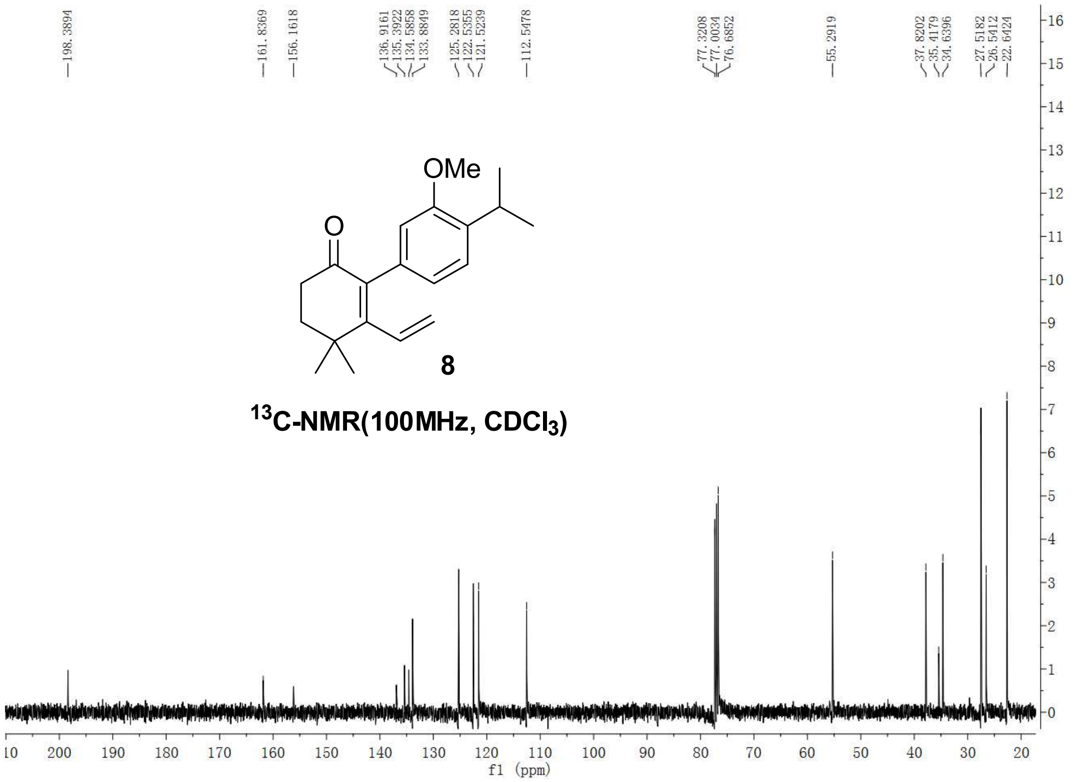

198.3894
161.8369
156.1618
136.9161
135.3922
134.5858
133.8849
125.2818
122.5355
121.5239
112.5478
77.3208
77.0034
76.6852
55.2919
37.8202
35.4179
34.6396
27.5182
26.5412
22.6424
OMe
O
8
^{13}C-NMR(100MHz, CDCl$_3$)
f1 (ppm)

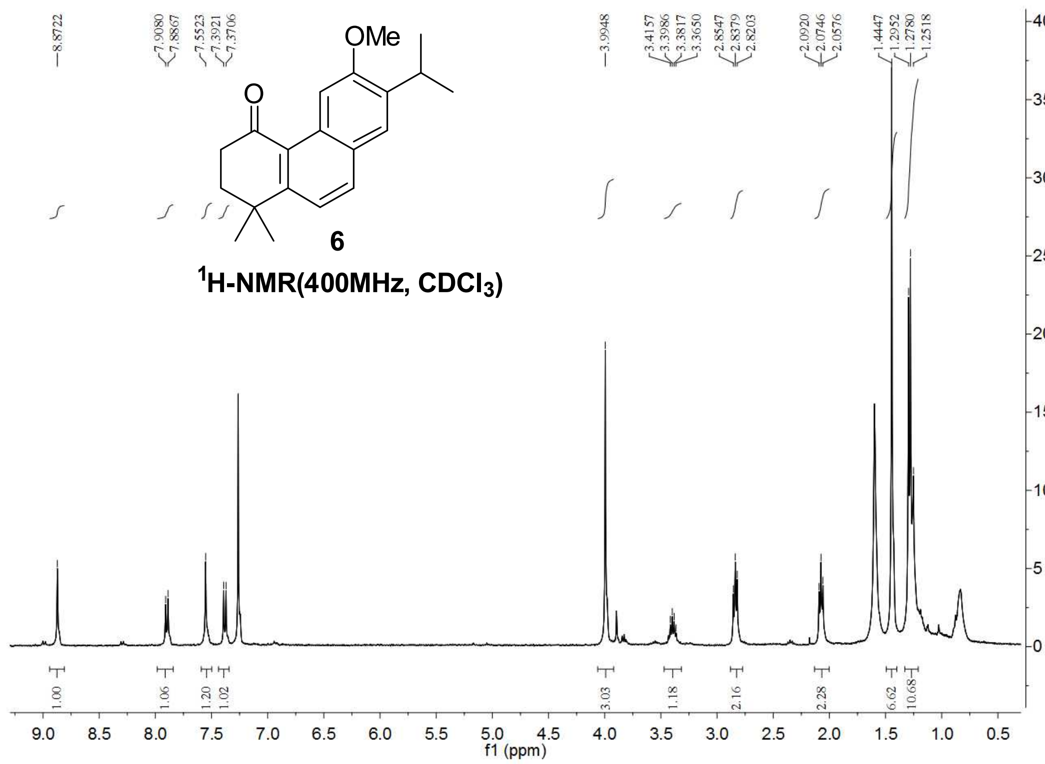
8.8722
7.9080
7.8867
7.5523
7.3921
7.3706
3.9948
3.4157
3.3986
3.3817
3.3650
2.8547
2.8379
2.8203
2.0920
2.0746
2.0576
1.4447
1.2952
1.2780
1.2518
OMe
O
6
1H-NMR(400MHz, CDCl3)
1.00
1.06
1.20
1.02
3.03
1.18
2.16
2.28
6.62
10.68
f1 (ppm)

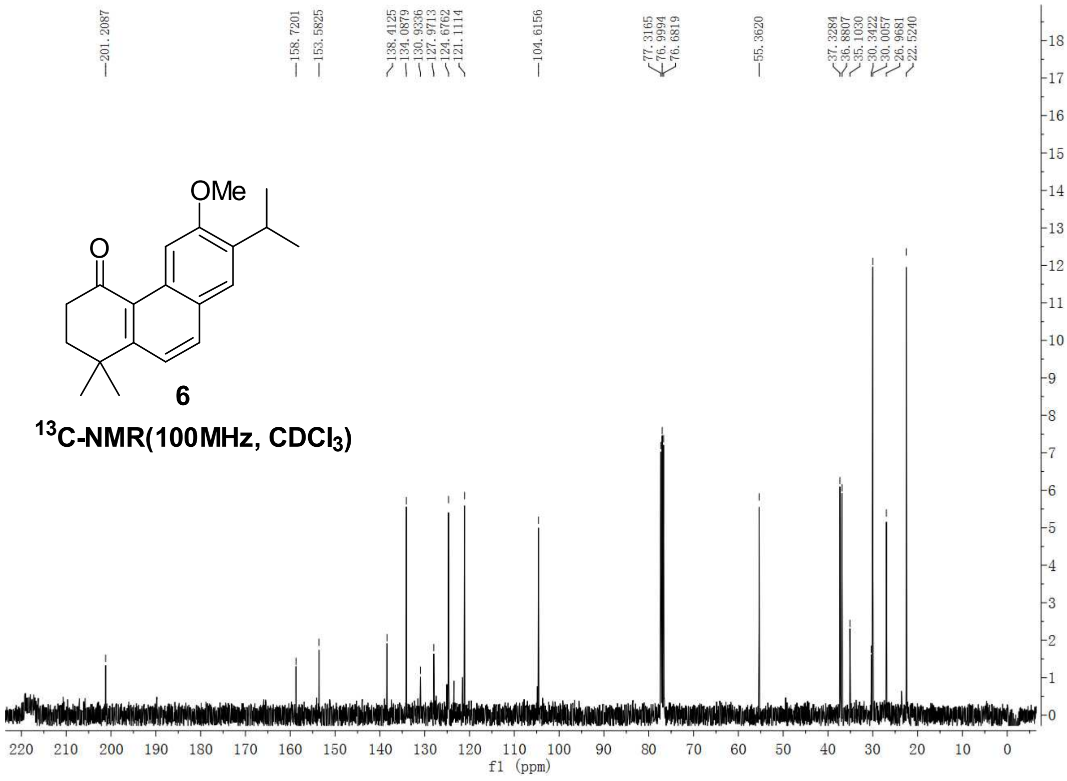
201. 2087
158. 7201
153. 5825
138. 4125
134. 0879
130. 9336
127. 9713
124. 6762
121. 1114
104. 6156
77. 3165
76. 9994
76. 6819
55. 3620
37. 3284
36. 8807
35. 1030
30. 3422
30. 0057
26. 9681
22. 5240
OMe
O
6
13C-NMR(100MHz, CDCl3)
f1 (ppm)

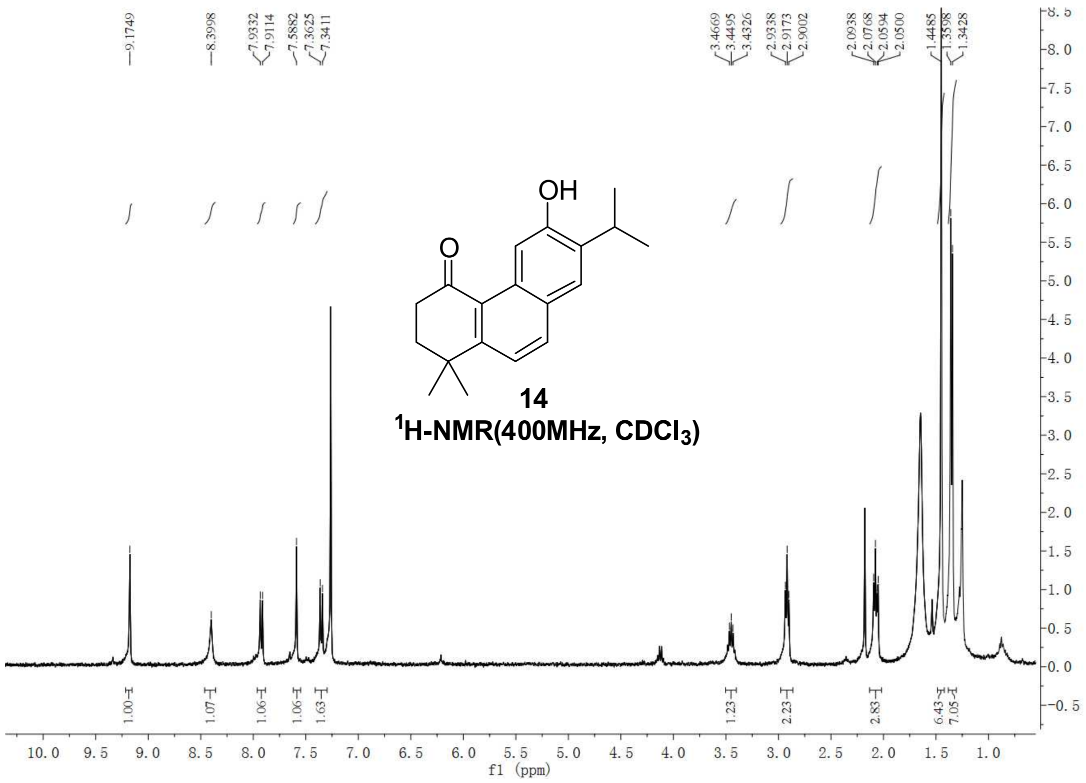

9.1749
8.3998
7.9332
7.9114
7.5882
7.3625
7.3411
3.4669
3.4495
3.4326
2.9338
2.9173
2.9002
2.0938
2.0768
2.0594
2.0500
1.4485
1.3598
1.3428
OH
O
14
1H-NMR(400MHz, CDCl3)
1.00
1.07
1.06
1.06
1.63
1.23
2.23
2.83
6.43
7.05
f1 (ppm)

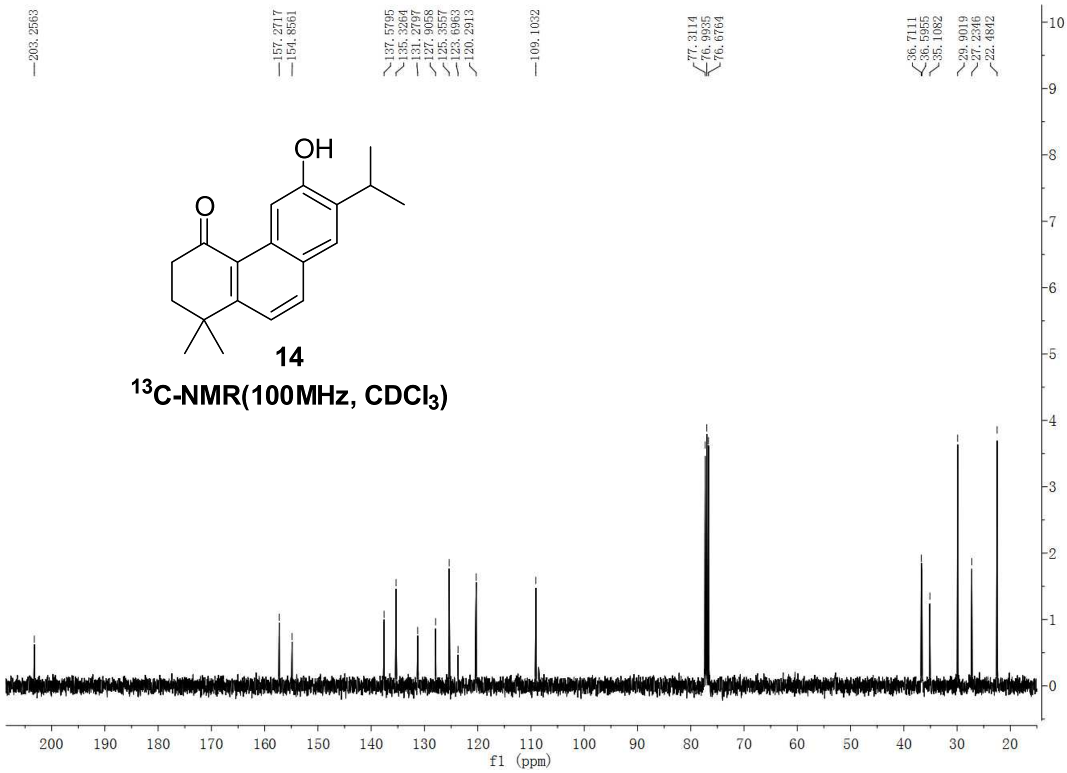

203.2563
157.2717
154.8561
137.5795
135.3264
131.2797
127.9058
125.3557
123.6963
120.2913
109.1032
77.3114
76.9935
76.6764
36.7111
36.5955
35.1082
29.9019
27.2346
22.4842
OH
O
14
13C-NMR(100MHz, CDCl3)
f1 (ppm)

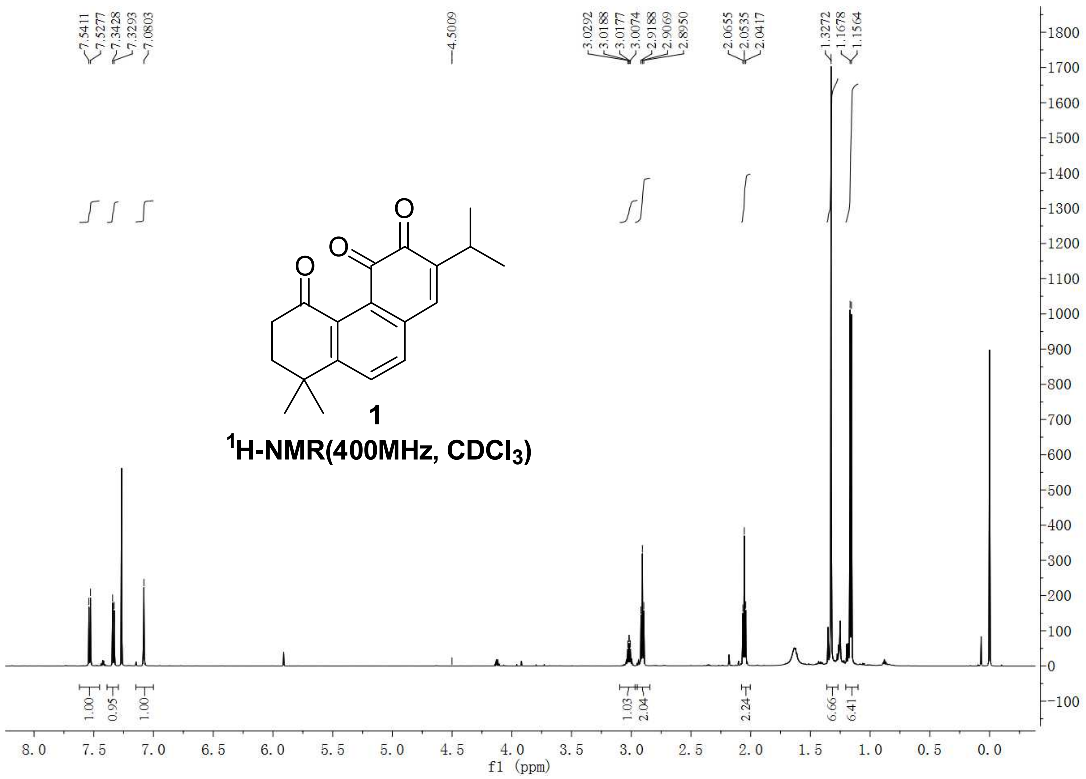
7.5411
7.5277
7.3428
7.3293
7.0803
4.5009
3.0292
3.0188
3.0177
3.0074
2.9188
2.9069
2.8950
2.0655
2.0535
2.0417
1.3272
1.1678
1.1564
O
O
O
1
1H-NMR(400MHz, CDCl3)
1.00
0.95
1.00
1.03
2.04
2.24
6.66
6.41
f1 (ppm)

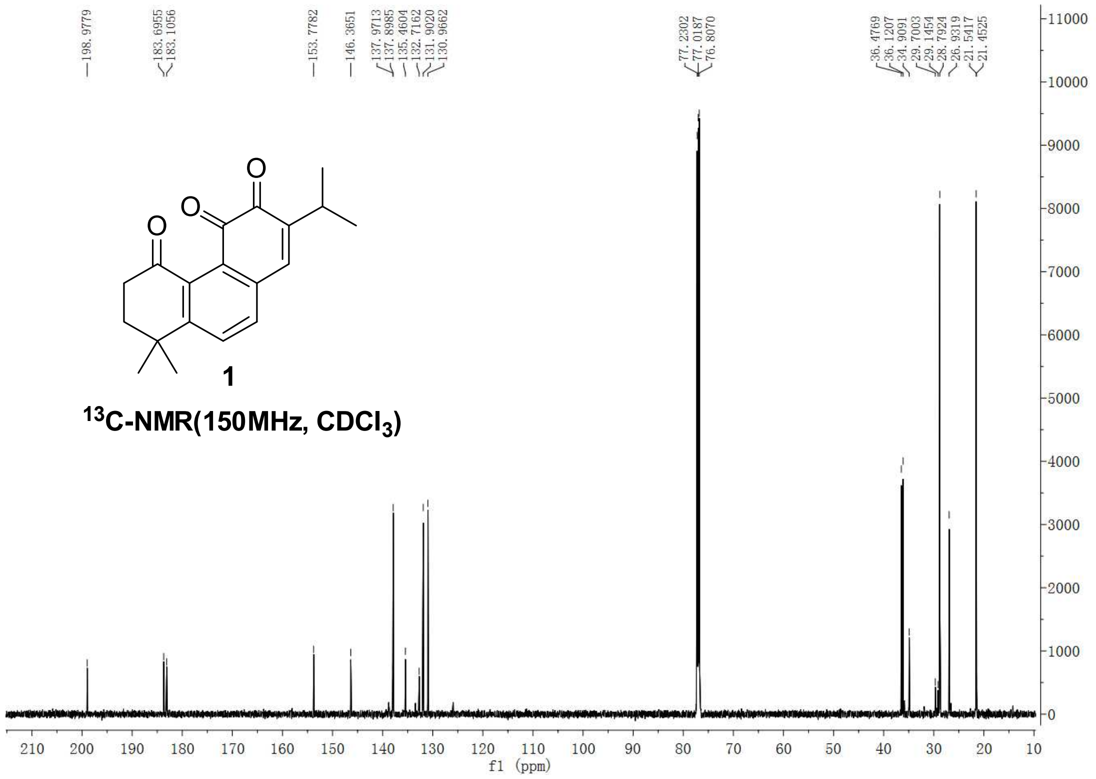
198.9779
183.6955
183.1056
153.7782
146.3651
137.9713
137.8985
135.4604
132.7162
131.9020
130.9662
77.2302
77.0187
76.8070
36.4769
36.1207
34.9091
29.7003
29.1454
28.7924
26.9319
21.5417
21.4525
O
O
O
1
13C-NMR(150MHz, CDCl3)
f1 (ppm)
